# CALLY is not associated with colorectal polyps risk: a systematic evaluation and mechanism exploration controlling for traditional factors and Traditional Chinese Medicine constitution

**DOI:** 10.3389/fpubh.2026.1953202

**Published:** 2026-10-21

**Authors:** Kui Zhang, Xiaokun Wu, Fang Lin, Boyuan Chen, Yubin Han

**Affiliations:** 1 Department of Gastroenterology, The Eighth Clinical Medical College of Guangzhou University of Chinese Medicine, Foshan, China; 2 Department of Gastroenterology, Foshan Hospital of Traditional Chinese Medicine, Foshan, China

**Keywords:** CALLY, colorectal polyps, risk factor, systematic evaluation, Traditional Chinese Medicine constitution

## Abstract

**Background:**

Globally, colorectal cancer (CRC) ranks as the third most common malignancy in terms of incidence. Colorectal polyps (CP) are the most common precancerous lesions of CRC, making the timely identification of their risk factors critically important. Although the CALLY index has theoretical promise as a tool for stratifying the risk of CP occurrence, its clinical utility remains unvalidated and warrants rigorous empirical assessment. Traditional risk factor models primarily focus on measurable biological indicators, often overlooking differences in an individual's overall functional state. Incorporating traditional Chinese medicine constitution (TCMC) is expected to enable the development of more comprehensive risk prediction models. Therefore, this study aims to validate whether CALLY is an independent risk factor for CP and to conduct preliminary exploration of its potential biological mechanisms, based on systematic control of traditional medical risk factors and TCMC.

**Methods:**

This study is a retrospective cross-sectional study that enrolled 452 hospitalized patients undergoing colonoscopy between October and December 2025. By constructing a stepwise-adjusted logistic regression model, conducting interaction analyses, testing mediating effects, and performing sensitivity analyses, we systematically investigated the relationship between CALLY and CP and its potential mechanisms.

**Results:**

Multivariate logistic regression analysis revealed no significant statistical association between CALLY quartile groups and CP risk (all comparisons *P* > 0.05). In contrast, male gender (OR: 0.56; 95%CI: 0.33–0.93; *P* < 0.05), advanced age (OR: 1.04; 95%CI: 1.02–1.06; *P* < 0.01), diabetes (OR: 3.82; 95%CI: 1.45–10.06; *P* < 0.01) and gastric polyps (GP, OR: 2.11; 95%CI: 1.26–3.53; *P* < 0.01) were consistently identified as independent risk factors. Regarding TCMC, the phlegm-dampness constitution showed a statistically significant but imprecise association (OR: 14.33; 95%CI: 3.60–57.05; *P* < 0.01). Mediation analysis further indicated that neither triglycerides nor high-density lipoprotein cholesterol played a significant mediating role in the association between CALLY and CP (*P*-values for direct, indirect, and total effects all > 0.05).

**Conclusions:**

Among hospitalized gastroenterology patients, CALLY is not an independent risk marker for CP and is not recommended as a routine screening indicator for this population for the time being. Risk assessment for such patients should focus on their gender, age, diabetes and gastric polyps. The phlegm-dampness constitution should be considered a preliminary finding due to the small subgroup size and wide confidence intervals; its role as a stable risk factor requires independent validation in larger prospective cohorts.

## Introduction

Globally, colorectal cancer (CRC) ranks as the third most common malignancy and is the second leading cause of cancer-related deaths (1). According to the 2022 Global Cancer Statistics, CRC accounted for approximately 9.6% of all new cancer cases worldwide, and CRC-related deaths represented 9.3% of all cancer deaths (2). In China, the incidence and mortality rates of CRC have shown a persistent upward trend. In 2022, approximately 520,000 new cases were diagnosed, with about 240,000 deaths occurring, making it a major public health issue (3). Colorectal polyps (CP) refer to protrusions extending from the typically flat mucosal surface of the colon into the lumen (4). They can be classified into adenomatous polyps and non-adenomatous polyps based on their histological characteristics and malignant potential (4). CP are the most common precancerous lesions of CRC (5, 6). Therefore, timely identification of risk factors for CP is crucial for reducing the incidence and mortality of CRC (7).

Previous studies have indicated that the risk of developing CP is closely associated with factors such as gender, age, body mass index (BMI), hypertension, diabetes, and Helicobacter pylori (HP) infection (8, 9). In recent years, the C-reactive protein-albumin-lymphocyte (CALLY) index proposed by the Iida research team in Japan has garnered widespread attention as a novel systemic inflammatory marker (10). This index integrates three key pathophysiological indicators common to tumors of the digestive system: C-reactive protein (CRP) reflecting systemic inflammation levels, albumin reflecting nutritional status, and absolute lymphocyte count (LYM) characterizing immune function (10). The CALLY index, as a composite biomarker, theoretically holds potential for assessing the risk of CP and may indirectly reflects the proliferation and differentiation of relevant cells as well as the patient's overall nutritional status (11). However, high-quality studies examining the association between the CALLY index and CP remain limited at present, and further research is needed to validate the clinical efficacy of the CALLY index in predicting CP.

It is worth noting that traditional risk factor models primarily focus on measurable biological indicators, often overlooking differences in an individual's overall functional state. Against this backdrop, Traditional Chinese Medicine's constitution theory offers a unique complementary perspective for risk assessment. As a vital component of fundamental theories in Traditional Chinese Medicine, this doctrine classifies individuals into nine primary constitutional types: Balanced Constitution, Qi Deficiency Constitution (QDC), Yang Deficiency Constitution (YDC), Yin Deficiency Constitution, Phlegm-Dampness Constitution (PDC), Damp-Heat Constitution (DHC), Blood Stasis Constitution (BSC), Qi Stagnation Constitution (QSC), and Special Constitution (12, 13). These constitutional classifications facilitate a holistic assessment of an individual's health status and provide healthcare professionals with a reference for identifying populations at high risk for potential diseases (14). Existing research indicates that specific Traditional Chinese Medicine constitutions (TCMC) are associated with the risk of developing and progressing colorectal adenomatous polyps. For example, the DHC is linked to an increased risk of colorectal adenoma progression (15). Relevant guidelines in China further clarify that PDC, DHC, and BSC are classified as high-risk TCMC for colorectal adenomatous polyps (16). Although domestic scholars have recognized the correlation between CP and TCMC, current research primarily focuses on the distribution characteristics of TCMC among CP patients. Comprehensive evaluations integrating the TCMC into CP prevention frameworks remain scarce, and explorations linking theory of traditional Chinese medicine to modern disease models are still relatively limited (16). Therefore, incorporating TCMC—a key variable reflecting an individual's overall functional state—into CP risk assessment holds promise for developing more comprehensive risk prediction models and more accurately evaluating the predictive value of the CALLY index for CP.

To date, no studies have systematically examined the association between the CALLY index and CP risk after adjusting for traditional risk factors and TCMC. This study aims to address this gap by conducting a rigorously designed observational study. For the first time, it will systematically evaluate the risk association between the CALLY index and CP within a framework that balances these two types of factors, and further explore its potential underlying mechanisms.

## Methods

### Research subjects

This retrospective cross-sectional study enrolled 452 patients hospitalized in the Department of Gastroenterology at a Grade III Class A traditional Chinese medicine hospital in China between October and December 2025 who underwent colonoscopy. Inclusion criteria for research subjects are: (1) age ≥18 years, regardless of gender; (2) completion of a colonoscopy; (3) complete medical records including clinical data, blood test results, and colonoscopy findings. Exclusion criteria are: (1) Severe organ dysfunction; (2) Pregnant or lactating women; (3) Concurrent malignant tumors or history of malignancy; (4) History of chemotherapy or radiotherapy; (5) Recent active infections, such as respiratory or urinary tract infections; (6) Confirmed chronic inflammatory or autoimmune diseases, including rheumatoid arthritis, systemic lupus erythematosus, or inflammatory bowel disease; (7) History of major trauma, surgery, or burns within the past 3 months; (8) Coagulation disorders or platelet abnormalities.

### Primary variable definitions

The exposure variable in this study is the CALLY index, calculated as CALLY = albumin (g/L) × LYM (10^9^/L) / CRP (mg/L) (17). In this study, all hematological examination items were collected from venous blood samples after the patients had fasting for 8 h and before intestinal preparation. Specimens were immediately transported to the laboratory for standardized testing.

The outcome variable of this study is CP. Each research subject underwent a colonoscopy and was classified into either the non-polyposis group or the polyposis group based on the colonoscopy findings. Prior to a colonoscopy, patients must follow dietary instructions: maintain a bland diet for 3 days, avoid consuming vegetables, fruits, and meat the day before the procedure, and begin fasting after dinner the night before the examination. All research subjects underwent a standardized bowel preparation protocol, which involved oral administration of 1 liter of polyethylene glycol electrolytes Powder (III) the night before the examination, followed by an additional 2 liters of the same solution within 2 h on the morning of the examination. If the research subject passed clear, residue-free bowel movements, bowel preparation was considered complete and colonoscopy could proceed. All colonoscopies were performed by experienced gastroenterologists following standardized colonoscopy procedures. According to the 2020 Consensus on pathological diagnosis of gastrointestinal adenoma and benign epithelial polyps (18), the diagnostic criteria for CP in this study were as follows: During colonoscopy, if a gastroenterologist observed a localized, protruding lesion arising from the surrounding colonic mucosa, and after endoscopic resection, the specimen was placed in a formalin-filled container and subjected to histopathological examination, a diagnosis of CP was established when the results revealed inflammatory polyps (not otherwise specified), colonic adenomas, or other benign epithelial polyps.

### Covariate definitions

The covariates in this study include age, gender, BMI, hypertension, diabetes, smoking and drinking status, TCMC, tongue color, tongue morphology, the properties of the tongue coating, tongue coating color, HP infection, gastric polyps (GP), gallbladder polyps, gallbladder stones, CRP, albumin, LYM, triglycerides (TG), and high-density lipoprotein cholesterol (HDL-C). Hypertension and diabetes were defined as conditions with a documented history prior to the research subject's admission, or as conditions clinically diagnosed by a physician during the current hospitalization. We categorized smoking and drinking status as either present or absent. The TCMC, tongue color, tongue shape, the properties of the tongue coating, and tongue coating color were all determined by experienced Chinese medicine practitioner based on fundamental TCM theory through the Eight Principles of Syndrome Differentiation for the subjects under study. TCMC and tongue parameters (color, morphology, and coating properties) were assessed by two experienced TCM practitioners (with more than 5 years of clinical experience in gastroenterology) who had undergone pre-study training and standardization on the classification criteria. Assessments were performed prior to colonoscopy, and the practitioners were blinded to all laboratory results (including CALLY and lipid parameters) and colonoscopy findings. In cases of diagnostic disagreement between the two practitioners, a third senior TCM practitioner (with >10 years of experience) was consulted, and the final classification was made after joint discussion and consensus. TCMC classified constitutions into categories including PDC, DHC, and BSC. Tongue color was classified into pale-dark, pale, pale-red, red, and dark-red. Tongue morphology was categorized into normal tongue, teeth-marked tongue, enlarged tongue, and fissured tongue. Tongue coating properties were described as thin, greasy, putrid, or thick. Tongue coating colors primarily included white and yellow. The presence of HP infection was determined by the C_13_ urea breath test, with a positive indicating infection and a negative indicating no infection. GP were diagnosed through gastroscopy and classified as present or absent. The determination of gallbladder polyps and gallstones was based on the results of abdominal ultrasound and abdominal CT scans provided by experienced radiologists, and was categorized as either present or absent.

### Statistical analysis

All statistical analyses were performed using R software version 4.2.2. Continuous variables following a normal distribution are described using the mean ± standard deviation (x ± S). Comparisons between two groups are performed using an independent samples *t*-test, while comparisons among multiple groups are conducted using analysis of variance (ANOVA). Continuous variables with non-normal distributions were represented as M (*P*25, *P*75) and compared using the Mann-Whitney U test or Kruskal-Wallis test. Categorical variables were described using frequency and percentage, and compared using the chi-square test. To comprehensively explore the relationship between CALLY and CP, we conducted a two-stage preliminary analysis: (1) Participants were divided into two groups based on the presence or absence of CP, and the distributions of CALLY and other variables were compared; (2) To avoid potential information loss from categorization, we initially treated CALLY as a continuous variable in the multivariate logistic regression. Variables with *P* < 0.10 in univariate analysis, together with CALLY (based on theoretical considerations), were entered into the initial full model. The backward likelihood ratio method was then applied for variable selection, with a removal criterion of *P* > 0.10. Model fit was assessed using the Hosmer-Lemeshow goodness-of-fit test. Additionally, to explore potential non-linear patterns that may have been overlooked by linear modeling, we performed a quartile-based sensitivity analysis as an alternative specification of CALLY. The resulting *P*-values of baseline comparisons between groups were descriptive. Given the exploratory nature of these analyses, no correction for multiple comparisons was applied.

To explore the independent association between CALLY and CP, we constructed three progressively adjusted binary logistic regression models: Model 1 was unadjusted for any variables; Model 2 adjusted for gender, age, BMI, diabetes, TCMC, tongue color, tongue morphology, and GP; Model 3 further adjusted for TG and HDL-C. Effect sizes for all models were expressed as odds ratios (OR) with corresponding 95% confidence intervals (95% CI). To evaluate effect modification across population subgroups regarding the CALLY - CP association, we added interaction terms to the logistic regression model (based on Model 2 covariates) and tested their significance. Mediation effect analysis employed the causal stepwise approach combined with the Bootstrap method. Bootstrap sampling with 5,000 repetitions was used to calculate robust standard errors and 95% CI for the path coefficients, to address the impact of non-normal variable distributions. If the 95% CI for the indirect effect did not include zero, the mediating effect was considered significant.

To assess the robustness of the main analytical conclusions, we conducted a sensitivity analysis. After excluding individuals with CALLY levels at the extreme ends (*P*5, *P*95), we fully replicated the analysis process of Model 2 to examine the impact of outliers on the core findings. All tests were performed using two-tailed tests, with *P* < 0.05 indicating statistically significant differences.

Finally, *post-hoc* power analysis was performed using PASS 15.0 with the logistic regression Wald test. Assuming an odds ratio of 1.5 for the highest vs. lowest CALLY quartile, with a two-sided alpha of 0.05, a sample size of 452, and an overall CP prevalence of 63%, the achieved statistical power was 41.6%. This indicates that the study was insufficiently powered to detect modest associations, and therefore, the null findings should be interpreted with caution and considered exploratory.

## Results

### Baseline characteristics of research subjects

This study included 452 subjects, comprising 289 patients with CP (63.27%) and 163 patients without CP (36.73%). Compared with non-CP patients, CP patients had a higher mean age (54.49 ± 12.17 years vs. 50.87 ± 13.51 years, *P* < 0.01) and a higher proportion of males (56.40% vs. 38.04%, *P* < 0.01), while the prevalence of diabetes (4.84% vs. 10.43%, *P* < 0.05) and the detection rate of GP (27.34% vs. 51.53%, *P* < 0.01) were significantly lower. Analysis of presenting symptoms showed that abdominal pain was significantly more common in the patients without CP than patients with CP (34.36% vs. 14.53%, *P* < 0.001), while abdominal distension did not differ significantly between them (9.82% vs. 6.57%, *P* = 0.20). The most prevalent TCMC among patients with CP was BSC (48.44%), with pale-red tongue color (40.48%) and normal tongue morphology (86.85%) being the most common findings. In contrast, among patients without CP, the predominant TCMC was QDC (52.76%), while pale-red tongue color (47.90%) and normal tongue morphology (85.90%) were also most frequently observed. Statistically significant differences were found between the two groups in terms of TCMC (*P* < 0.01), tongue color (*P* < 0.01), and tongue morphology (*P* < 0.05). Please see Table 1 for details.

**Table 1:** Comparison of baseline characteristics among research subjects grouped by presence of CP.

Variables	CP group (*n* = 289)	Non-CP group (*n* = 163)	Test statistic	*P* value
Age (year), x ± s	54.49 ± 12.17	50.87 ± 13.51	*t* = −2.84	<0.01^**^
BMI (kg/m^2^), M (*P*25, *P*75)	23.33 (20.77, 25.16)	22.70 (20.44, 25.53)	Z = −0.71	0.48
CRP (mg/L), M (*P*25, *P*75)	1.61 (0.49, 4.69)	1.45 (0.50, 4.69)	Z = −0.16	0.87
Albumin (g/L), M (*P*25, *P*75)	43.43 (42.50, 45.15)	43.43 (42.20, 45.60)	Z = −0.52	0.60
LYM (10^9^/L), M (*P*25, *P*75)	1.75 (1.43, 2.15)	1.76 (1.35, 2.17)	Z = −0.46	0.65
TG (mmol/L), M (*P*25, *P*75)	1.34 (0.91, 1.78)	1.23 (0.90, 1.66)	Z = −1.16	0.25
HDL-C (mmol/L), M (*P*25, *P*75)	1.38 (1.21, 1.47)	1.38 (1.21, 1.55)	Z = −1.71	0.09
CALLY, M(*P*25, *P*75)	46.24 (17.48, 159.17)	48.96 (16.93, 150.85)	Z = −0.04	0.97
Gender, *n*(%)			χ^2^ = 14.06	<0.01^**^
Male	163 (56.40%)	62 (38.04%)		
Female	126 (43.60%)	101 (61.96%)		
Symptom, *n*(%)				
Abdominal pain	42 (14.53%)	56 (34.36%)	χ^2^ = 22.96	<0.01^**^
Abdominal distension	19 (6.57%)	16 (9.82%)	χ^2^ = 1.61	0.20
Hypertension, *n*(%)	64 (22.15%)	27 (16.56%)	χ^2^ = 2.02	0.16
Diabetes, *n*(%)	14 (4.84%)	17 (10.43%)	χ^2^ = 5.09	0.02^*^
Smoking, *n*(%)	16 (5.54%)	3 (1.84%)	χ^2^ = 3.54	0.06
Drinking, *n*(%)	14 (4.84%)	4 (2.45%)	χ^2^ = 1.56	0.21
TCMC, *n*(%)			χ^2^ = 121.48	<0.01^**^
QSC	14 (4.84%)	26 (15.95%)		
BSC	140 (48.44%)	7 (4.29%)		
PDC	12 (4.15%)	8 (4.91%)		
QDC	50 (17.30%)	86 (52.76%)		
DHC	64 (22.15%)	30 (18.40%)		
YDC	9 (3.12%)	6 (3.69%)		
Tongue color, *n*(%)			χ^2^ = 19.44	<0.01^**^
Pale-dark	77 (26.64%)	16 (9.80%)		
Pale-white	32 (11.07%)	25 (15.30%)		
Pale-red	117 (40.48%)	78 (47.90%)		
Pale	24 (8.30%)	18 (11.00%)		
Red	36 (12.46%)	22 (13.50%)		
Dark-red	3 (1.05%)	4 (2.50%)		
Tongue morphology, *n*(%)			χ^2^ = 0.013	0.046^*^
Normal	251 (86.85%)	140 (85.90%)		
Teeth-marked	16 (5.54%)	14 (8.60%)		
Enlarged	7 (2.42%)	2 (1.20%)		
Fissured	15 (5.19%)	7 (4.30%)		
Tongue coating property, *n*(%)			χ^2^ = 1.74	0.63
Thin	238 (82.35%)	141 (86.50%)		
Greasy	48 (16.61%)	21 (12.90%)		
Putrid	1 (0.35%)	0 (0.00%)		
Thick	2 (0.69%)	1 (0.60%)		
Tongue coating color, *n*(%)			χ^2^ = 0.14	0.71
White	228 (78.89%)	131 (80.40%)		
Yellow	61 (21.11%)	32 (19.60%)		
HP, *n*(%)	29 (10.03%)	16 (9.82%)	χ^2^ = 0.01	0.94
GP, *n*(%)	79 (27.34%)	84 (51.53%)	χ^2^ = 26.47	<0.01^**^
Gallbladder polyps, *n*(%)	18 (6.23%)	6 (3.68%)	χ^2^ = 1.35	0.25
Gallstones, *n*(%)	15 (5.19%)	11 (6.75%)	χ^2^ = 0.47	0.49

^*^P < 0.05; ^**^P < 0.01.

BMI, CRP, albumin, LYM, TG, and HDL-C exhibited statistically significant differences across CALLY quartile groups (*P* < 0.05). As CALLY levels increased, BMI and CRP levels showed a decreasing trend (*P* < 0.01). See Table 2 for details. CALLY did not show statistically significant differences between the CP group and the non-CP group (*P* = 0.97). Similarly, there were no significant statistical differences in CP prevalence across CALLY quartile groups (*P* = 0.76).

**Table 2:** Comparison of baseline characteristics among research subjects grouped by CALLY quartiles.

Variables	Q1 (*n* = 113)	Q2 (*n* = 113)	Q3 (*n* = 113)	Q4 (*n* = 113)	*P-*value
Age (year), M (*P*25, *P*75)	55.00 (47.00, 67.00)	53.00 (46.50, 62.50)	55.00 (43.00, 60.00)	52.00 (41.50, 60.00)	0.12
BMI (kg/m^2^), M (*P*25, *P*75)	23.78 (21.28, 26.60)	23.88 (21.68, 26.11)	22.84 (20.52, 24.98)	21.48 (19.83, 23.88)	<0.01^**^
CRP (mg/L), M (*P*25, *P*75)	4.69 (4.69, 9.16)	3.01 (1.77, 4.69)	0.95 (0.61, 1.34)	0.25 (0.09, 0.44)	<0.01^**^
Albumin (g/L), M (*P*25, *P*75)	43.43 (40.40, 44.15)	43.43 (43.20, 45.30)	43.43 (42.30, 46.15)	43.43 (42.80, 45.65)	<0.01^**^
LYM (10^9^/L), M (*P*25, *P*75)	1.53 (1.28, 1.80)	1.84 (1.45, 2.20)	1.80 (1.41, 2.24)	1.85 (1.55, 2.29)	<0.01^**^
TG (mmol/L), M (*P*25, *P*75)	1.40 (0.92, 1.82)	1.31 (0.96, 1.92)	1.41 (0.94, 1.79)	1.08 (0.81, 1.66)	<0.05^*^
HDL-C (mmol/L), M (*P*25, *P*75)	1.38 (1.13, 1.43)	1.38 (1.20, 1.48)	1.38 (1.23, 1.57)	1.38 (1.28, 1.60)	<0.01^**^
Gender, *n*(%)					0.99
Male	55 (24.4%)	57 (25.3%)	57 (25.3%)	56 (24.9%)	
Female	58 (25.6%)	56 (24.7%)	56 (24.7%)	57 (25.1%)	
Hypertension, *n*(%)	30 (33.0%)	23 (25.3%)	21 (23.1%)	17 (18.7%)	0.18
Diabetes, *n*(%)	11 (35.5%)	6 (19.4%)	5 (16.1%)	9 (29.0%)	0.39
Smoking, *n*(%)	7 (36.8%)	2 (10.5%)	6 (31.6%)	4 (21.1%)	0.37
Drinking, *n*(%)	6 (33.3%)	4 (22.2%)	4 (22.2%)	4 (22.2%)	0.93
TCMC, *n*(%)					0.37
QSC	10 (25.0%)	9 (22.5%)	10 (25.0%)	11 (27.5%)	
BSC	32 (21.8%)	39 (26.5%)	33 (22.4%)	43 (29.3%)	
PDC	4 (20.0%)	8 (40.0%)	7 (35.0%)	1 (5.0%)	
QDC	29 (21.3%)	34 (25.0%)	36 (26.5%)	37 (27.2%)	
DHC	32 (34.0%)	20 (21.3%)	24 (25.5%)	18 (19.1%)	
YDC	6 (40.0%)	3 (20.0%)	3 (20.0%)	3 (20.0%)	
Tongue color, *n*(%)					0.28
Pale-dark	24 (25.8%)	24 (25.8%)	24 (25.8%)	21 (22.6%)	
Pale-white	18 (31.6%)	16 (28.1%)	9 (15.8%)	14 (24.6%)	
Pale-red	44 (22.6%)	48 (24.6%)	47 (24.1%)	56 (28.7%)	
Pale	6 (14.3%)	15 (35.7%)	15 (35.7%)	6 (14.3%)	
Red	18 (31.0%)	9 (15.5%)	17 (29.3%)	14 (24.1%)	
Dark-red	3 (42.9%)	1 (14.3%)	1 (14.3%)	2 (28.6%)	
Tongue morphology, *n*(%)					0.46
Normal	100 (25.6%)	94 (24.0%)	99 (25.3%)	98 (25.1%)	
Teeth-marked	4 (13.3%)	12 (40.0%)	7 (23.3%)	7 (23.3%)	
Enlarged	1 (11.1%)	2 (22.2%)	4 (44.4%)	2 (22.2%)	
Fissured	8 (36.4%)	5 (22.7%)	3 (13.6%)	6 (27.3%)	
Tongue coating property, *n*(%)					0.36
Thin	90 (23.7%)	99 (26.1%)	96 (25.3%)	94 (24.8%)	
Greasy	23 (33.3%)	14 (20.3%)	16 (23.2%)	16 (23.2%)	
Putrid	0 (0.0%)	0 (0.0%)	0 (0.0%)	1 (100%)	
Thick	0 (0.0%)	0 (0.0%)	1 (33.3%)	2 (66.7%)	
Tongue coating color, *n*(%)					0.65
White	86 (24.0%)	93 (25.9%)	88 (24.5%)	92 (25.6%)	
Yellow	27 (29.0%)	20 (21.5%)	25 (26.9%)	21 (22.6%)	
HP, *n*(%)	12 (26.7%)	13 (28.9%)	14 (31.1%)	6 (13.3%)	0.30
GP, *n*(%)	35 (21.5%)	46 (28.2%)	45 (27.6%)	37 (22.7%)	0.32
CP, *n*(%)	70 (24.2%)	76 (26.3%)	69 (23.9%)	74 (25.6%)	0.76

^*^P < 0.05; ^**^P < 0.01.

### Multifactorial association analysis between CALLY and CP

To comprehensively evaluate the strength and independence of the association between CALLY and CP, this study divided CALLY into four groups (Q1–Q4) based on quartile ranges and constructed three progressive multivariate logistic regression models for analysis. Based on Model 1 results, using the lowest quartile group (Q1) of CALLY as the reference, no statistically significant association was observed between CP and the remaining quartile groups (Q2, Q3, Q4) (all comparisons *P* > 0.05). The specific OR and their 95% CI are detailed in Table 3. To further control for potential confounding effects, we adjusted for gender, age, BMI, diabetes, TCMC, tongue color, tongue morphology, and GP. According to the results of Model 2, there remained no significant association between the CALLY quantile groups and CP (all comparisons *P* > 0.05). Further adjusting for TG and HDL-C based on Model 2, the results showed that the association between CALLY quantiles and CP remained non-significant (all comparisons *P* > 0.05). Notably, in both Model 2 and Model 3, risk factors consistently and stably associated with CP include: male gender (*P* < 0.05), advanced age (*P* < 0.01), diabetes (*P* < 0.01), TCMC (particularly PDC, *P* < 0.01), and GP (*P* < 0.01). The OR and 95% CI are detailed in Table 3.

**Table 3:** Multivariate logistic regression analysis of factors associated with CP.

Variables	Model 1		Model 2		Model 3	
	OR (95%CI)	*P-*value	OR (95%CI)	*P-*value	OR (95%CI)	*P-*value
CALLY		0.74		0.81		0.84
Q1	1 (reference)	-	1 (reference)	-	1 (reference)	-
Q2	0.86 (0.50–1.48)	0.58	0.80 (0.40–1.63)	0.54	0.81 (0.39–1.67)	0.57
Q3	1.08 (0.62–1.88)	0.78	1.06 (0.52–2.18)	0.87	1.03 (0.49–2.14)	0.94
Q4	0.83 (0.48–1.42)	0.49	0.82 (0.41–1.63)	0.57	0.81 (0.41–1.61)	0.54
Gender	-	-	0.56 (0.33–0.93)	0.03^*^	0.57 (0.33–0.97)	0.04^*^
Age	-	-	1.04 (1.02–1.06)	<0.01^**^	1.04 (1.02–1.06)	<0.01^**^
Diabetes	-	-	3.82 (1.45–10.06)	<0.01^**^	3.90 (1.47–10.34)	<0.01^**^
TCMC				<0.01^**^		<0.01^**^
QSC	-	-	1 (reference)	-	1 (reference)	-
BSC	-	-	0.29 (0.08–1.14)	0.08	0.32 (0.08–1.25)	0.10
PDC	-	-	14.33 (3.60–57.05)	<0.01^**^	15.50 (3.82–62.98)	<0.01^**^
QDC	-	-	1.01 (0.21–4.84)	0.99	1.02 (0.21–5.01)	0.98
DHC	-	-	0.47 (0.13–1.62)	0.23	0.49 (0.14–1.72)	0.26
YDC	-	-	1.04 (0.27–4.03)	0.95	1.10 (0.28–4.31)	0.89
GP	-	-	2.11 (1.26–3.53)	<0.01^**^	2.10 (1.25–3.52)	<0.01^**^
BMI	-	-	0.98 (0.91–1.05)	0.56	0.97 (0.90–1.04)	0.43
Tongue color				0.93		0.93
Pale-dark	-	-	1 (reference)	-	1 (reference)	-
Pale-white	-	-	1.24 (0.16–9.56)	0.84	1.16 (0.15–8.93)	0.89
Pale-red	-	-	1.09 (0.14–8.52)	0.94	1.01 (0.13–7.95)	0.99
Pale	-	-	1.64 (0.23–11.62)	0.62	1.55 (0.22–10.96)	0.66
Red	-	-	1.27 (0.16–10.36)	0.83	1.21 (0.15–9.86)	0.86
Dark-red	-	-	1.49 (0.19–11.61)	0.70	1.37 (0.18–10.70)	0.76
Tongue morphology				0.37		0.30
Normal	-	-	1 (reference)	-	1 (reference)	-
Teeth-marked	-	-	0.48 (0.15–1.53)	0.22	0.48 (0.15–1.54)	0.22
Enlarged	-	-	0.28 (0.06–1.21)	0.09	0.25 (0.06–1.09)	0.07
Fissured	-	-	0.82 (0.08–8.44)	0.87	0.80 (0.08–7.88)	0.85
TG	-	-	-	-	1.14 (0.92–1.41)	0.24
HDL-C	-	-	-	-	0.96 (0.41–2.24)	0.92

^*^P < 0.05; ^**^P < 0.01. Model 2: AUC = 0.85, Hosmer-Lemeshow test *P*-value was 0.90; Model 3: AUC = 0.85, Hosmer-Lemeshow test *P-*value was 0.98.

### Interaction analysis

Building upon Model 2, this study conducted an interaction analysis to further investigate whether the effect of CALLY on CP varies according to gender, age, diabetes, TCMC, and GP. Interaction analysis revealed that the association pattern between the CALLY quartile groups and CP (which did not reach statistical significance in this study) showed no significant alterations across different genders, ages, diabetes statuses, TCMC, or GP (*P* > 0.05). Please see Table 4 for details.

**Table 4:** Interaction test for CALLY and CP.

Item	Wald χ^2^ value	df	*P-*value
CALLY quartile groups ^*^ Gender	5.90	3	0.12
CALLY quartile groups ^*^ Age	3.27	3	0.35
CALLY quartile groups ^*^ Diabetes	1.84	3	0.61
CALLY quartile groups ^*^ TCMC	9.01	15	0.88
CALLY quartile groups ^*^ GP	3.83	3	0.28

All models were adjusted for gender, age, BMI, diabetes, TCMC, tongue color, tongue morphology, and GP. CALLY was divided into quartiles Q1–Q4, with Q1 as the reference group. Interaction terms were tested using the likelihood ratio test (LR test).

### Sensitivity analysis

To assess the robustness of our main findings, we conducted several sensitivity analyses.

First, to rule out the possibility that the quartile categorization obscured a linear dose-response relationship, we repeated the multivariate logistic regression analysis with CALLY entered as a continuous variable. In the initial full model containing 11 variables, the backward selection procedure sequentially removed tongue color, chronic atrophic gastritis, smoking, chronic non-atrophic gastritis, tongue morphology, and CALLY. CALLY was not retained in the final model, showing no significant association with CP (OR = 1.000, 95% CI: 1.000–1.001, *P* = 0.22), with a negligible contribution to model explanatory power (ΔR^2^ = 0.003). The final model was statistically significant (χ^2^ = 2.45, *P* < 0.01) and demonstrated good calibration (Hosmer-Lemeshow *P* = 0.96, Nagelkerke R^2^ = 0.43). This continuous-variable analysis further confirms that CALLY does not exhibit a linear association with CP risk, consistent with the quartile-based findings.

Second, to exclude the possibility of a non-linear or threshold relationship that might have been missed by both the quartile-based and linear continuous analyses, we further performed a restricted cubic spline (RCS)-equivalent sensitivity analysis using natural splines with 3 degrees of freedom (equivalent to 4 knots) in the fully adjusted model (Model 2 covariates). The likelihood ratio test comparing the spline model with the linear model showed no significant evidence of non-linearity (χ^2^ = 0.25, Δdf = 2, *P* = 0.881), indicating that the spline terms did not improve model fit. This result further confirms that CALLY does not exhibit a substantial non-linear or threshold association with CP risk in this cohort.

Third, sensitivity analyses excluding tongue signs or GP from Model 2 yielded results consistent with the main findings, with all CALLY quartile groups remaining non-significantly associated with CP (*P* > 0.05 for all comparisons). Detailed results are provided in Supplementary Table S1. To examine the robustness of the main conclusions, we conducted the analysis again after excluding observations with CALLY scores falling in the top and bottom 5% extremes (*P*5 = 4.67, *P*95 = 1,024.57). After adjusting for the same covariates as Model 2 (gender, age, BMI, diabetes, TCMC, tongue color, tongue morphology, GP), the sensitivity analysis results were highly consistent with the main analysis: (1) No significant statistical association remained between CALLY quartile groups and CP (all comparisons *P* > 0.05); (2) Male sex (*P* < 0.05), advanced age (*P* < 0.01), diabetes (*P* < 0.01), TCMC (particularly PDC, *P* < 0.01), and GP (*P* < 0.01) remained independent risk factors for CP in the sensitivity analysis model. Please see Supplementary Table S2 for details.

Collectively, these sensitivity analyses were supporting the robustness of our conclusion that CALLY is not independently associated with CP risk in hospitalized gastroenterology patients.

### Exploratory analysis

To investigate whether CALLY influences CP by affecting lipid metabolism (TG or HDL-C), we conducted a mediation analysis. The analysis covers two potential pathways: “CALLY → TG → CP” and “CALLY → HDL-C → CP”. As shown in Table 5, after adjusting for gender, age, BMI, diabetes, TCMC, tongue color, tongue morphology, and GP: (1) In the “CALLY → TG → CP” pathway, CALLY was not significantly associated with TG (β = −0.001; 95% CI: −0.003 to 0.001; *P* = 0.37); TG also showed no significant association with CP (β = 0.13; 95% CI: −0.04 to 0.47; *P* = 0.31). (2) Similarly, in the “CALLY → HDL-C → CP” pathway, no significant associations were found: CALLY was not associated with HDL-C (β = −0.001; 95% CI: −0.002 to 0.001; *P* = 0.47), nor was HDL-C associated with CP (β = 0.12; 95% CI: −1.09 to 0.75; *P* = 0.77). (3) In the models including the mediating variables, the direct effect of CALLY on CP was not significant (*P* = 0.25 and *P* = 0.28 for the two pathways, respectively); the total effect of CALLY on CP was also non-significant (β ≈ 0.00; 95% CI: −0.001 to 0.001; *P* = 0.28). In summary, we did not find statistical evidence supporting a mediating role of TG or HDL-C.

**Table 5:** Mediating effect analysis of CALLY on CP via TG and HDL-C.

Effect type	Pathway	β coefficient	Standard error	95%CI	*P-*value
Indirect effects	CALLY → TG	<−0.01	<0.01	<−0.01 to <0.01	0.37
Indirect effects	TG → CP	0.13	0.11	−0.04 to 0.47	0.31
Direct effects	CALLY → CP	<0.01	<0.01	<−0.01 to <0.01	0.25
Indirect effects	CALLY → HDL-C	<−0.01	<0.01	<−0.01 to <0.01	0.47
Indirect effects	HDL-C → CP	−0.12	0.42	−1.09 to 0.75	0.77
Direct effects	CALLY → CP	<0.01	<0.01	<−0.01 to <0.01	0.28
Overall effect	CALLY → CP	<0.01	<0.01	<−0.01 to <0.01	0.28

β coefficient represents the unstandardized regression coefficient.

## Discussion

This study employed a cross-sectional design to systematically evaluate the association between serum CALLY and CP and its underlying mechanisms. Through the construction of a stepwise adjustment model, interaction and mediation analyses, and after fully controlling for traditional factors and TCM constitution, consistent findings indicate that the CALLY index is not an independent influencing factor for CP. This conclusion remains robust across different models and sensitivity analyses.

A recent study of individuals undergoing health screenings found that higher CALLY scores were associated with lower CP risk, with this association being more pronounced in women (7). This study reached different conclusions, and such discrepancies may stem from variations in the nature of the study populations and the influence of key confounding factors. The subjects of this study were hospitalized patients in the Department of Gastroenterology, whose clinical backgrounds were more complex. Unlike general health-screening cohorts, the majority of our participants underwent colonoscopy due to gastrointestinal symptoms rather than routine screening. Notably, abdominal pain was significantly more frequent in the non-CP group than in the CP group (34.36% vs. 14.53%, *P* < 0.001). This finding suggests that our inpatient cohort represents a clinically enriched population with a higher baseline burden of gastrointestinal complaints, rather than a true screening population. In this cohort, we observed a significant comorbidity between GP and CP (27.34%, *P* < 0.01). Furthermore, in multivariate analysis, GP emerged as a strong independent risk factor for CP (OR: 2.11; 95% CI: 1.26–3.53; *P* < 0.01). This finding aligns with multiple Chinese studies, all of which confirm the co-occurrence of GP and CP, with the former being an independent risk factor for the latter (OR: 1.61; 95% CI: 1.05 to 2.46 for hyperplastic Polyps; OR: 1.45; 95% CI: 1.09 to 1.94 for fundic gland Polyp) (19, 20). In contrast, Li et al.'s study (7) based on a health screening cohort showed a lower GP prevalence and no significant confounding effect (11.22%, *P* = 0.079). Therefore, the heterogeneity of the population may directly lead to differences in the structure of confounding factors. In clinical settings where GP exhibit high prevalence and constitute a strong risk factor themselves, GP may become a significant confounding variable that distorts or masks the true association between CALLY and CP if not rigorously adjusted for. We failed to replicate the risk association of CALLY after rigorously controlling for gastric polyps. This suggests that the relationship between CALLY and CP may be highly dependent on the baseline risk characteristics of the study subjects. In clinical settings where GP coexist, CALLY has not yet been found to have independent predictive value. Therefore, in the risk assessment and management of CP among this population, routine testing for CALLY may have limited clinical utility. However, this conclusion should not be directly extrapolated to asymptomatic community screening populations without prospective validation.

This study also revealed a key contradiction: although CALLY showed a negative correlation with the lipid marker TG (*r* = −0.12, *P* = 0.01) and a positive correlation with HDL-C (*r* = 0.18, *P* < 0.01), exploratory analysis indicated that lipid metabolism pathways may not be an effective mediating pathway for CALLY to influence CP. To understand this phenomenon, we further analyzed the relationship between CALLY and lipid profile indicators. In the field of metabolic medicine, although the direct association between CALLY and lipid profiles remains unclear, its three components exhibit significant and independent links to lipid metabolism. For example, albumin serves as a nutritional indicator, exhibiting a negative correlation with TG (*r* = −0.276, *P* < 0.01) and a positive correlation with HDL-C (*r* = 0.381, *P* < 0.01) (21, 22). CRP is an inflammatory marker that shows a positive correlation with TG (*r* = 0.86, *P* < 0.01) (23) and a negative correlation with HDL-C (*r* = −0.360, *P* < 0.01) (24). Therefore, CALLY, as a composite indicator, may serve as an integrated signal reflecting an individual's overall metabolic inflammatory background rather than an active pathogenic factor that directly regulates blood lipids and subsequently leads to mucosal lesions.

Furthermore, this study provides substantial support for the empirical application of TCM theory in modern epidemiological research on CP. In all multivariate models, TCMC (particularly PDC) was also identified as a potential risk factor for CP, but the wide confidence intervals (OR 14.33, 95% CI: 3.60–57.05) indicate limited precision due to the small number of PDC cases in the study sample. This finding should be interpreted cautiously and requires independent validation in larger cohorts. PDC is characterized by a damp, heavy, and thick bodily condition. Individuals with this constitution often exhibit overweight, a greasy complexion, sluggish metabolism, and a predisposition to mucous accumulation (25). Modern research indicates that this constitution is closely linked to metabolic disorders such as obesity, hypertriglyceridemia, and non-alcoholic fatty liver disease (25–27). These disorders themselves are known risk factors for CP (28). The results of this study further suggest that the long-term, systemic functional imbalance tendencies reflected by TCMC may represent a more fundamental and stable layer of risk determination. Therefore, integrating TCMC identification (particularly the assessment of PDC) into clinical risk screening and primary prevention strategies for CP can serve as a valuable supplement to existing risk assessment systems. Future research should further elucidate the precise molecular pathways through which PDC promotes the occurrence of CP, and validate whether personalized interventions based on constitutional regulation can effectively reduce the risk of CP. This will advance the practical translation of integrated Chinese and Western medicine prevention strategies.

## Conclusions

This study demonstrates through systematic evaluation that after fully adjusting for traditional factors and TCMC, the CALLY is not an independent risk factor for CP, and no mediating pathway through lipid metabolism (TG/HDL-C) was identified. Additionally, the study confirmed that male gender, advanced age, diabetes, and GP were stable and independent risk factors. The phlegm-dampness constitution, while showing a statistically significant association, should be considered a preliminary finding due to the small subgroup size and wide confidence intervals. This finding provides empirical evidence for establishing a CP risk assessment system that integrates both Chinese and Western medical influencing factors.

## Limitations

This study has several limitations that should be considered when interpreting the findings. First, the cross-sectional, single-center design limits both causal inference and generalizability; the observed associations, or lack thereof, should be extrapolated to other populations with caution, and future multi-center prospective cohort studies are needed to confirm these observations. Second, both the CALLY index and lipid parameters were measured only once, which may introduce measurement error due to individual biological variability and could attenuate true associations. Third, several established risk factors for CP—including detailed dietary habits, physical activity levels, family history of colorectal cancer or polyps, and use of anti-inflammatory medications—were not available in this retrospective dataset, and residual confounding from these unmeasured variables cannot be fully excluded.

Furthermore, given the limited sample size and the consequent reduction in statistical power, the null findings from our exploratory analyses should be interpreted with caution; they cannot definitively rule out small or moderate mediating effects, particularly when further stratified analyses were precluded by insufficient subgroup sizes. The absence of a significant overall association between CALLY and CP in this study does not necessarily negate the potential clinical relevance of this marker, but rather highlights the need for adequately powered studies to better characterize its role.

## Data Availability

The original contributions presented in the study are included in the article/Supplementary material, further inquiries can be directed to the corresponding author.
