# Supplementary material for: CALLY is not associated with colorectal polyps risk: a systematic evaluation and mechanism exploration controlling for traditional factors and Traditional Chinese Medicine constitution
Source: Front Public Health. 2026 Oct 21;14:1953202. doi: 10.3389/fpubh.2026.1953202 (PMC13731402; doi:10.3389/fpubh.2026.1953202)
Supplement: Supplementary file 1 [file Table_1.docx]

Table S1. Sensitivity Analyses for the Association Between CALLY Quartiles and CP

| Model | CALLY Q2 vs Q1 | CALLY Q3 vs Q1 | CALLY Q4 vs Q1 |
| --- | --- | --- | --- |
| Primary Model 2 | OR=0.80, *P*=0.54 | OR=1.06, *P*=0.87 | OR=0.82, *P*=0.57 |
| Sensitivity A (excluding tongue signs) | OR=0.79, *P*=0.51 | OR=1.00, *P*=1.00 | OR=0.82, *P*=0.56 |
| Sensitivity B (excluding GP) | OR=0.80, *P*=0.53 | OR=1.01, *P*=0.97 | OR=0.79, *P*=0.50 |

| Table S2. Sensitivity Analysis of the Association Between Cally and CP | | | | | | |
| --- | --- | --- | --- | --- | --- | --- |
| Variables | β value | SE | Wald χ² value | *P* | OR | 95%CI |
| Constant | -2.78 | 1.78 | 2.44 | 0.12 | 0.06 | - |
| **Cally** |  |  | 0.53 | 0.91 |  |  |
| Q2 | 0.24 | 0.38 | 0.39 | 0.53 | 1.27 | 0.60-2.68 |
| Q3 | 0.04 | 0.39 | 0.01 | 0.92 | 1.04 | 0.49-2.21 |
| Q4 | 0.01 | 0.37 | ＜0.01 | 0.97 | 1.01 | 0.49-2.08 |
| Gender | 0.69 | 0.28 | 6.07 | 0.01* | 0.5 | 0.29-0.87 |
| Age | 0.04 | 0.01 | 11.15 | ＜0.01** | 1.04 | 1.02-1.06 |
| Diabetes | 1.4 | 0.52 | 7.08 | ＜0.01** | 4.04 | 1.44-11.28 |
| TCMC |  |  | 52.23 | ＜0.01** |  |  |
| QSC | -1.01 | 0.72 | 1.96 | 0.16 | 0.37 | 0.09-1.50 |
| BSC | 2.9 | 0.73 | 15.82 | ＜0.01** | 18.19 | 4.36-75.95 |
| PDC | 0.36 | 0.84 | 0.18 | 0.67 | 1.43 | 0.28-7.36 |
| QDC | -0.51 | 0.65 | 0.63 | 0.43 | 0.6 | 0.17-2.13 |
| DHC | 0.17 | 0.72 | 0.06 | 0.81 | 1.19 | 0.29-4.83 |
| YDC | 0.77 | 0.28 | 7.87 | ＜0.01** | 2.17 | 1.26-3.72 |
| BMI | -0.02 | 0.04 | 0.32 | 0.57 | 0.98 | 0.91-1.06 |
| Tongue color |  |  | 1.91 | 0.86 |  |  |
| Pale-white | 0.8 | 1.22 | 0.43 | 0.51 | 2.22 | 0.21-24.04 |
| Pale-red | 0.57 | 1.23 | 0.21 | 0.65 | 1.76 | 0.16-19.52 |
| Pale | 0.91 | 1.17 | 0.6 | 0.44 | 2.48 | 0.25-24.68 |
| Red | 0.49 | 1.24 | 0.16 | 0.69 | 1.63 | 0.14-18.55 |
| Dark-red | 0.62 | 1.22 | 0.26 | 0.61 | 1.86 | 0.17-20.07 |
| Tongue morphology |  |  | 2.19 | 0.54 |  |  |
| Teeth-marked | -0.68 | 0.61 | 1.25 | 0.26 | 0.51 | 0.16-1.67 |
| Enlarged | -1.07 | 0.77 | 1.94 | 0.16 | 0.34 | 0.08-8.75 |
| Fissured | -0.14 | 1.18 | 0.02 | 0.9 | 0.87 | 0.09-8.75 |
| Note:*P＜0.05；**P＜0.01. | | | | | | |
